# Honey Origin Authentication via Mineral Profiling Combined with Chemometric Approaches

**DOI:** 10.3390/foods12152826

**Published:** 2023-07-25

**Authors:** Anže Pavlin, Drago Kočar, Jernej Imperl, Mitja Kolar, Gregor Marolt, Petranka Petrova

**Affiliations:** 1 Faculty of Chemistry and Chemical Technology, University of Ljubljana, Večna pot 113, SI-1000 Ljubljana, Slovenia; anze.pavlin@fkkt.uni-lj.si (A.P.); drago.kocar@fkkt.uni-lj.si (D.K.); jernej.imperl@fkkt.uni-lj.si (J.I.); gregor.marolt@fkkt.uni-lj.si (G.M.); 2 Faculty of Mathematics and Natural Sciences, South-West University “Neofit Rilski”, Ivan Mihailov, 66, 2700 Blagoevgrad, Bulgaria

**Keywords:** chestnut, honey, minerals, microwave digestion, ICP-MS, multielement analysis, PCA, origin

## Abstract

In the present study, the potential of elemental analysis combined with statistical tools to identify honey origin was evaluated by mineral characterization of 173 honeys of 13 floral types (acacia, fir, spruce, linden, chestnut, lavender, coriander, thistle, honeydew, rosemary, sage, euphorbia and ziziphus plant species) collected from five geographical regions (Slovenia, Croatia, Bulgaria, Turkey, and Morocco). The objective of the study was to accurately and reliably differentiate the mineral composition among honey varieties. The aim was to establish traceability, to ensure product authenticity and to improve quality control measures within the honey industry. For this purpose, 18 major, minor and trace elements were quantified using microwave digestion, followed by ICP-MS measurement. Statistical evaluation of elemental concentration was undertaken using principal component analysis (PCA) to distinguish honey floral types. The research give light on the specific elements that can serve as indicators for determining the geographical and botanical source of honey. Our findings indicate that certain elements, such as Mn, K, and Ca, are primarily influenced by the type of pollen present in the honey, making them indicative of the floral source. On the other hand, levels of Na, Mg, and Fe were found to be more strongly influenced by environmental factors and can be considered as markers of geographical origin. One novel aspect of this research is the exploration of the relationship between honey minerals and honey botanical source. This was achieved through the analysis of chestnut tree samples and a subsequent comparison with the composition of chestnut honey.

## 1. Introduction

Honey, propolis, royal jelly, beebread, venom, and wax are all products synthesized by bees, with complex compositions traditionally used for human nutrition and health. Among them, honey is characterized by specific and variable chemical content determined by its origin, both geographical and botanical, environmental conditions, as well as by processing procedures.

In general, honey contains mostly sugars. Bogdanov [1] identified more than 25 carbohydrates, establishing that fructose and glucose are the major sugar constituents [2]. Honey also comprises substances in micro and trace quantities, which produce numerous nutritional and biological effects. It contains enzymes, proteins, amino and organic acids, vitamins, lipids, flavonoids, and phenolic acids. The mineral elements are also among the minor constituents of honey. A survey of the literature shows that the total mineral content of honey is between 0.1% and 0.2% for blossom honeys and almost 1% for honeydew honeys [3,4]. The water content in honey is usually below 20%.

Although present in small quantities, the minor constituents determine honey’s biological activity and therapeutic properties. Different studies have proved various physiological effects resulting from honey consumption that include antimicrobial, antiviral and antiparasitic activity, and antioxidant and anti-inflammatory effects. Honey has a positive effect on the gastrointestinal and cardiovascular systems. Furthermore, the oligosaccharides in honey stimulate the growth and activity of lactobacilli and bifidobacteria and exert a prebiotic effect [5,6,7]. According to some reports, honey positively influences alcohol-induced liver damage and gastric lesions, and decreases alcohol concentration in the blood [8].

Though the main components of honey are almost identical, the type and the quantity of minor constituents determine its odor, taste, color, and all perceptual properties. The taste and color depend on the non-volatile compounds like amino acids, minerals, and phenolic compounds, whereas the aroma of honey is mainly dependent on the volatile and semi-volatile components [9].

The rich variety of plant species, which are pollen and nectar sources for bee food, different geographical origins and seasonal conditions determine the great diversity in honey appearance, sensory qualities, and nutritional and health effects [4]. Some authors emphasize that the botanical source has a more dominant effect on the chemical composition of the honey compared with the geographical factors, specifying that botanical origin differentiates honey samples better than geographical provenance [3,10].

Depending on the plant substrate used by bees, two types of honey can be distinguished: honeydew produced from secretions of living parts of plants or excretions of plant-sucking insects, and blossom honey produced from the nectar of flowers. They differ in their chemical composition and physicochemical properties, giving specific color, aroma, taste, and health benefits. Furthermore, blossom honey can be classified as monofloral or polyfloral, depending on the pollen content of different plant species. Monofloral honey is produced from one plant species, containing predominantly its nectar and pollens. It is rare that honey is 100% of one type of flower. Usually, it is a mixture with a predominance (>45%) of one type of flower species. Polyfloral honey, known also as multifloral, has several botanical sources where none is predominant. In general, monofloral honeys are regarded as more valuable products; however, the polyfloral honeys are more commercially available. 

One way to ascertain the type of honey is melissopalynology, the analysis of pollen contained in honey, which can determine its floral origin according to the methodology introduced by [11]. This technique quantifies the dominant pollen in the honey, allowing the honey to be named after the botanical species of the plant. Because the method is time consuming and requires qualified specialists, melissopalynology is being replaced by the physio-chemical analysis of the honey combined with statistical methods. The current approach involves quantifying parameters such as color, sugar content, pH, moisture, phenolic and flavonoid compounds, and amino acids [12,13,14,15]. Besides these, minerals are good marker for honey type because they reflect the local conditions and can be indicative of the plant and regional origin [14]. Natural and anthropogenic factors influence the mineral composition of honey. For example, metals can be introduced from the dust coming from soils and rocks, as described in the literature [16,17,18]. Other sources of metals in honey are human activities resulting in air, water, soil, and plant pollution, suggesting that bee products can be used as potential bioindicators for background pollution [19,20,21].

Given the large amounts of data gathered when analyzing many honey samples, statistical analysis becomes a suitable tool to better understand the acquired data. Multivariate techniques such as principal component analysis (PCA) allow classification of samples by some characteristic or property. PCA analysis is widely used when dealing with characterization and authentication of foodstuffs, e.g., wines [22,23,24]. In the case of honey, clustering based on the mineral composition can distinguish honeys by their geographical or floral provenance [25,26,27,28]. Elemental analysis of Slovenian honeys of different floral varieties served as the dataset used for chemometric evaluation of possible differentiation of honeys according to their botanical origin. 

Considering that the honey’s origin is reflected in its mineral composition, the elemental analysis can be used to differentiate and identify honey types. This study includes the quantification of number of elements associated with litho- or pedogenic sources, such as aluminium, calcium, iron, and magnesium, and some toxic elements, such as arsenic, cadmium, nickel, and lead. The aim was to distinguish natural from anthropogenic metal sources in honey composition, as well as to investigate the potential presence of toxic elements exerting a significant health hazard.

However, a review of the existing literature revealed that honey has received little attention in the study of certain anthropogenic elements such as Pt, Pd, and Rh.

In addition, the relationship between the composition of honey and the composition of the plants that bees use as food has been poorly studied. Based on our data, there is currently no information on the minerals in honey compared with the minerals in the corresponding plant sources.

To enhance the current knowledge, we undertook comprehensive research on honeys with specific geographic and botanical sources. 

Primarily, these studies focused on exploring the relationship between the mineral composition and the origin of the honey.

Secondly, we aimed to expand the existing knowledge on anthropogenic contaminants in honey, including the analysis of platinum group metals. 

Finally, our third goal was to better understand the cycling of elements in nature by analyzing the influence of plant precursors on honey composition.

Through these studies, we aimed to improve the general understanding of how plant source composition, environmental conditions, and human activities influence the mineral composition of honey.

For this purpose, honeys from widespread botanical species such as acacia, linden, and chestnut, as well as honeys from little studied plants such as *Euphorbia* sp. and *Ziziphus lotus* L., were included in the study [29]. 

To achieve these objectives, we employed the ICP-MS technique, which enables simultaneous multi-element measurements while maintaining remarkably low detection limits. Additionally, ICP-MS stands as an excellent technique with its high sensitivity and wide linear range. ICP-MS offers simple spectra allowing for the determination of both major and trace elements. 

## 2. Materials and Methods

### 2.1. Sample Collection

The present study involved the analysis of 173 honey samples including monofloral, polyfloral, and honeydew honeys collected from Slovenia, Croatia, Bulgaria, Turkey, and Morocco. 

One hundred and forty-two honey samples from seven botanical origins, namely, acacia (26), mixed flower or floral (60), forest (28), fir (2), spruce (2), linden (18), and chestnut (6), were collected from seven municipalities in Slovenia between 2016 and 2018. The sampling area covered all regions of Slovenia. This gives the possibility of comparing the honey composition according to the honey type. All honey samples were obtained from the company Medex and local beekeepers.

Eighteen honey samples from salvia, rosemary and mixed flower types were harvested from Croatian islands, including sixteen honeys from Hvar (12 floral, 2 sage, and 2 rosemary), one honey from Vis (floral), and one honey sample from Ščedro (rosemary) for assessment of the background levels of trace metals in honey from remote areas.

Six monofloral honeys from lavender, coriander, acacia, linden, thistle, honeydew, and two polyfloral honeys were purchased from Bulgarian manufacturers. 

Tree polyfloral honeys were purchased from different regions of Turkey, namely, Yüksekova, Kayseri, and Muş. 

The two samples from Morocco were Euphorbia honey (*Euphorbia* sp.) and Ziziphus honey (*Ziziphus lotus* L.). Euphorbia is a plant in the spurge family Euphorbiaceae, which includes several species. *Ziziphus lotus* L. (*Z. lotus*) is from the family Rhamnaceae called Sedra. There are more than 40 species of the genus *Ziziphus*. In the Mediterranean region, the plant is represented by *Z. lotus* species [30]. 

All honeys from Croatia, Bulgaria, Turkey, and Morocco were collected in the spring and summer of 2022. The botanical origins of the studied honeys were determined by beekeepers based on the availability of floral sources at the hive location. Until the time of analysis, all samples were stored in a dark place in the laboratory at room temperature. The geographical and botanical characteristics of the studied honey samples are summarized in Table 1.

Leaves, flowers, and cupules (fruit husks) of a chestnut tree *Castanea sativa* from Slovenia were collected and analyzed for elemental content to establish the possible correlation between the elemental composition of honey with that of the plant species. 

### 2.2. Reagents

All reagents used for the analysis were of analytical reagent grade. Nitric (V) acid (65%) obtained from LabExpert and hydrogen peroxide (30%) purchased from Fluka were used for honey digestion. Ultrapure (MilliQ) water (resistivity > 18.2 MΩ cm^−1^, produced from Millipore/MilliQ system) was used to prepare all solutions. A multi-element stock standard solution (Periodic table mix 1 for ICP), 10 mg L^−1^, containing 33 elements was purchased from Sigma Aldrich^®^. The working standard solutions were prepared when needed through appropriate dilutions with freshly prepared 1% HNO_3_ solution in ultrapure (MilliQ) water. 

### 2.3. Sample Preparation

The honey samples were accurately weighed (approximately 0.5 g) into PTFE vessels, and 8 mL of concentrated HNO_3_ and 2 mL of 30% H_2_O_2_ solution were added. The vessels were closed and placed in the microwave digestion system. A digestion program for honey samples was selected from the manual of the microwave system with the following temperature program: 25 min ramp to 210 °C and 20 min hold at 210 °C. (The power was set to 1800 W).

After digestion, the PTFE vessels were left to cool to room temperature. The samples were quantitatively transferred into 50 mL volumetric flasks and filled up to the mark with ultrapure (MilliQ) water. The honey samples were digested in duplicates. Blank samples were prepared with the same procedure.

The combination of HNO_3_ and H_2_O_2_ with microwave digestion is often used by researches to ensure the complete solubilization of honey samples [31,32,33,34,35,36].

The chestnut tree samples (leaves, flowers, and cupules) were lyophilized and were prepared with the same procedure as the honey samples.

### 2.4. Instruments

Sample digestion was conducted in a high-pressure microwave oven (Milestone Ethos UP, Italy). Quantification of the studied elements in acid digested honey samples was performed using an ICP-MS spectrometer (Agilent 7900, Agilent Technologies, Santa Clara, CA, USA) under optimal instrumental parameters ensuring the lowest detection limits.

The digested samples were diluted five- and fifty-times and used for measurements of selected elements.

Due to its unique advantages, ICP-MS is becoming increasingly common in honey analysis [32,33,34,35,36]. An optimization of the instrumental parameters for ICP-MS measurements was performed so that lowest detection limits were achieved. The specifications of the spectrometer and the operating conditions are presented in Table 2.

A standard calibration using seven standard solutions was performed, paying attention to closely matching the matrix of standards and blanks with the samples in terms of acid concentration and pre-treatment. 

The analytical precision was assessed by triplicate measurements of the signals and was expressed as standard deviation (SD) from the mean value. For elements with high concentrations, the SDs were under 3%, and for elements with lower concentrations, they were under 10%. The method accuracy was verified by analyte addition tests using three concentration levels, reaching recoveries from 85 to 110% with RSD below 10%.

### 2.5. Statistical Analysis

All the analyzed honey samples were measured in triplicate and the mean values of the analyses are presented. For the honeys collected in Slovenia, a descriptive statistic for the major, minor, and trace elemental concentration is presented in Tables S1 and S2. The summarized data include a measure of the central tendency, expressed as mean value, and a measure of the variability, expressed as standard deviation, relative standard deviation, minimum and maximum values. The descriptive statistic presenting the concentration range and dispersion of the mineral content for a large number of honeys of the same type could be informative for assessing the variability and distribution of mineral levels within a particular type of honey. 

One hundred and thirty-eight well-defined Slovenian honey samples with known geographical and botanical origin were collected, and numerous samples of the same floral type were analyzed, thus allowing the use of multivariate statistical methods. Elemental profiles (B, Mg, Al, K, Mn, Fe, Ni, Cu, Zn, Rb, Sr, and Ba) of the Slovenian honeys (acaciaSi, chestnutSi, lindenSi, floralSi, and forestSi) were used for PCA analysis to distinguish the honey types. The data were standardized, and using PCA analysis, the score and loading plots of the first two principal components were graphed. The matrix size for the PCA analysis includes 138 samples of Slovenian honey of 5 different botanical origins. 12 elements were assessed. The PCA analysis was performed with Origin (OriginPro 2018 (v9.50)). 

To assess the significant differences in the concentration of the major elements (K, Ca, Na and Mg) as well as in the total mineral content among all the honey samples examined, a Duncan test was performed using SPSS (IBM STATISTICS 26, Armonk, NY, USA) software. The results were considered significant at *p* < 0.05. 

## 3. Results and Discussion

### 3.1. Mineral Composition

A total of 173 honey samples with variety of botanical origin collected from different geographical areas were analyzed for 18 elements including major, minor, trace, and noble metals. The summarized results obtained for the studied elements for all honey samples are presented in Table S3.

**Figure 1:**
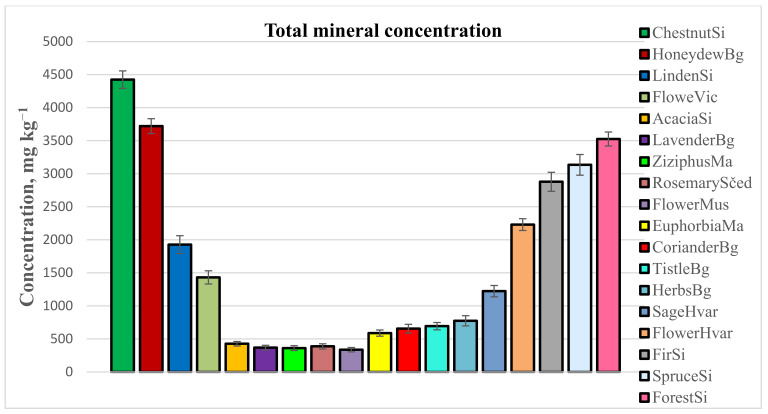
Total mineral content (mg kg^−1^) determined in studied honeys with various botanical and geographical origins.

The data revealed significant fluctuations in the total elemental concentration between the analyzed honey varieties. We can distinguish honeys with high mineralization, such as chestnutSi (4423.37 ± 3% mg kg^−1^), firSi (2878.26 ± 5% mg kg^−1^), and forestSi (3525.84 ± 3% mg kg^−1^) honeys, along with honeydewBg (3704.6 ± 3% mg kg^−1^) and spruceSi (3134.02 ± 5% mg kg^−1^). Considering several reports estimating honey composition, such high mineral levels are typical also for the carob [37], heather [26], avocado [38], and sesame [39] honeys. All these honeys are characterized not only by a high potassium level but also by a high percentage of the other major elements, such as calcium, sodium, and magnesium. In the present study, we established that honeys such as acaciaBg, lavenderBg, and ziziphusMa contained low total elemental concentrations of 268 ± 9% mg kg^−1^, 368 ± 10% mg kg^−1^, and 362 ± 10% mg kg^−1^, respectively, which are comparable to other light honeys reported in literature, such as citrus [40] and rosemary [37]. From the studied honeys, lindenSi (1927 ± 7% mg kg^−1^) showed intermediate mineralization. The obtained results in our study are similar to those previously reported by [41,42,43,44]. Figure 1 depicts the variations in the total mineral concentration resulting from different botanical and geographical origins of the honeys.

From the data summarized in Table S3, we can observe that the mineral profile of honey samples shows a wide differentiation. The concentrations of elements can vary greatly from country to country and from plant to plant, which correlates with past investigations performed on the Hungarian, Spanish, Polish, New Zealand, and Moroccan honeys listed in Table S4. In general, the variation in the mineral content is more pronounced for the major elements, such as potassium, sodium, and calcium, compared with the minor and trace elements. 

In our research, the highest concentration values were measured for potassium, which is reported to be the most abundant mineral in honey around the world, although there is a significant variation in its concentration. Expressed as a percentage of the total mineral content, K levels in most of the studied honeys were between 82% and 97%. Chudzinska and Baralkiewicz [45] discovered that K content depends on the botanical origin, and this finding was confirmed in our study. We established the largest K content in chestnutSi, firSi, forestSi, spruceSi and honeydewBg honeys, with concentrations between 2720 and 4204 mg kg^−1^. However, there were honeys with relatively low K content, such as ziziphus, euphorbia, lavender, acacia, and thistle (Figure S1), as found in some other reports. Low K content was noted by Rashed and Soltan [39] in clover honey, with 20% K along with 45% for Ca and 28% for Mg, which shows that there are honeys where K is not the dominate element. Kaygusuz et al. [46] found an approximately equal content of K and Ca in lavender honey at 50% and 49%, respectively. 

Besides potassium, we established that the other major elements in honey were calcium, magnesium, and sodium. The lowest Ca concentration was found in rosemaryŠčed (2.0 ± 10% mg kg^−1^), acaciaBg (2.7 ± 10% mg kg^−1^), acaciaSi (5.4 ± 9% mg kg^−1^), and lavenderBg (6.0 ± 10% mg kg^−1^) All of these were honeys with low total mineral concentration. Although the Ca levels in the tested honeys were generally low, some of the investigated honeys were distinguished by a higher Ca content, such as 36.6 ± 10% mg kg^−1^ in chestnutSi honey (Figure S2). Similar levels of Ca content in Italian chestnut honeys were identified by Grembecka and Szefer [41]. However, significantly higher Ca concentrations were detected by Escuredo et al. [47] in Spanish chestnut honeys (159 mg kg^−1^), by Sajtos et al. [3] in Hungary chestnut honey (161 mg kg^−1^), and by Kaygusuz et al. [46] in Turkish chestnut honey (104 mg kg^−1^). These findings could be explained by specific local soil composition. It is worth noting that several independent factors influence the concentrations of Ca and K in honey. Considering that the plants are the major source of minerals in honey, soil chemistry and the depth of the root system of the plants have a significant role for potassium and calcium accumulation. Furthermore, the concentrations of mineral elements like calcium and potassium depend on the bioavailability and consequently vary in honeys from different botanical origins. On the other hand, the pollens of different botanical species have highly varying concentrations of mineral elements [48]. Thus, geographical, geological, and botanical factors influence K and Ca levels in honey. 

Generally, magnesium was found in a relatively narrow concentration range between 1.5 and 5.4% of the total mineral content in most of the studied samples, with the lowest concentrations in acaciaSi and acaciaBg honeys (8.7 ± 10% and 6.8 ± 10% mg kg^−1^) and the highest values in forestSi (110 ± 3% mg kg^−1^), and spruceSi (105 ± 3% mg kg^−1^) (Figure S3). ChestnutSi, ziziphusMa, and euphorbiaMa honeys contained medium Mg levels of 34.3–58.8 mg kg^−1^. This hierarchy of Mg levels in different plant species could be observed in the investigations of Vanhanen et al. [15], Chakir et al. [37], Atanassova et al. [49], Grembecka and Szefer [41], and Sajtos et al. [3]. However, although the order is the same, the Mg content in honeys from different countries can be quite variable, which suggests an additional influence of geographical origin on the distribution of this element in honeys. This finding is not surprising considering that magnesium is one of the main elements in soils and rocks. 

In our work, we registered Na content in a broad concentration range from 2.4 ± 10% mg kg^−1^ in firSI honey to 142 ± 3% mg kg^−1^ in ziziphusMa honey (Figure S4). Chudzinska and Baralkiewicz [45] established that the Na content is comparable in all studied honey samples, regardless of honey’s botanical origin. As a result of our investigations, we can add that Na levels are comparable for different botanical types which are harvested from close localities. For example, we registered comparable Na concentrations for all Bulgarian types of honeys (between 9 and 16 mg kg^−1^), which was different from the Na level in the Turkish (19–36 mg kg^−1^) and Moroccan (108–142 mg kg^−1^) honeys. The observed high Na content in Moroccan honeys (ziziphus and euphorbia) may be related to proximity to the Mediterranean Sea, which is a source of salts in aerosols. This leads to increased mineral content in plants and soils. Therefore, based on our work, we can conclude that Na levels are more subject to geographical origin than to botanical influences, making it challenging to distinguish honeys from different plant species according to sodium data. It is particularly noticeable for the ziziphusMa and euphorbiaMa honeys where the high Na and Mg content is accompanied by low K levels, which was observed also by Chakir et al. [37], Latifa et al. [50], Bettar et al. [51], and Bouhlali et al. [52]. The tested Turkish polyfloral honeys showed a similar tendency of elevated Na and Mg levels and low values of K, but to a lesser extent. In this context, Stocker et al. [48] and Balkanska et al. [53] calculated K/Na and Mg/Ca ratios as a marker of seasonal and geographical variation in royal jelly.

Among the minor elements, iron and manganese were present in almost all the studied honeys. Iron is a soil-related element, and it is evident from the literature survey that its content in honey differs significantly from region to region. We can see from Table S4 that Egyptian and Malaysian honeys are distinguished by high Fe content ranging between 80 and 202 mg kg^−1^, in contrast to Bulgarian, Hungarian, New Zealand, Turkish, and Polish honeys in which this element is present in the range of 0.5–3.0 mg kg^−1^. The Moroccan and Spanish honeys have slightly higher Fe levels of 7–8 mg kg^−1^. Considering these findings, it is worth comparing the Fe content in honeys from different botanical species but with close geographical origins. In this context, Atanassova et al. [49] reported Fe values in Bulgarian honeys—acacia (0.8 mg kg^−1^), linden (1.6 mg kg^−1^), and coriandrum (1.3 mg kg^−1^)—that are comparable to those obtained for the same types of Bulgarian honey in the present study (0.5 mg kg^−1^ for acaciaBg, 1.7 mg kg^−1^ for lindenBg, and 1.0 mg kg^−1^ for corianderBg). 

Additionally, Tuzen et al. [54] studied trace element content in honeys from different regions of Turkey and measured the Fe concentration in honey from Kayseri province at 2.3 mg kg^−1^, which is very similar to the Fe content in Kayseri honey measured in this study (2.2 mg kg^−1^). 

**Figure 2:**
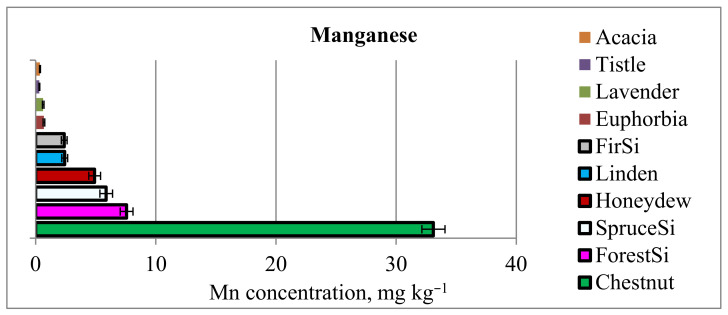
Concentrations of Mn (mg kg^−1^) in honeys with different plant origins.

Another mineral of interest is manganese. The content of Mn in various types of honey varied significantly. In most of the honeys, the Mn level was below 1 mg kg^−1^, with the lowest concentrations in ziziphusMa, acaciaBg, and corianderBg honeys in the range of 0.1–0.2 mg kg^−1^. Considerably higher concentrations of Mn were measured in forestSi (7.6 ± 5% mg kg^−1^), spruceSi (5.9 ± 7% mg kg^−1^), and honeydewBg (4.9 ± 9% mg kg^−1^) honeys. However, these levels are distinctly lower than the concentrations detected in chestnutSi honey, in which the Mn content reached 33.1 ± 3% mg kg^−1^ (Figure 2). This corresponds to previously reported findings for the highest Mn concentration in chestnut, linden, and honeydew honey, regardless of their geographical origin [3,41,49,55]. 

Regarding Mn levels, some investigations have demonstrated that oak and eucalyptus honeys are also characterized by elevated levels of this element [26,46,56]. Rodríguez-Flores et al. [57] pointed out that the physicochemical characteristics attributed to oak honey have very similar patterns to those in chestnut honey. In this sense, it is possible to suggest that manganese is one of the elements which is a more specific marker for botanical species than for geographical origin. 

The essential elements copper, zinc, chromium, and cobalt were present at lower concentration levels as trace elements. Among them, copper was detected in all the samples except ziziphusMa with relatively constant concentrations from 0.02 to 0.06% of the elemental profile of honey. 

In our research, we observed that Zn levels were more pronounced in polyfloral honeys from Kayzeri and Muş provinces in Turkey, with values of 3.5 ± 7% mg kg^−1^ and 3.8 ± 7% mg kg^−1^, respectively. Higher Zn levels can be caused by anthropogenic activity. Demirezen and Aksoy [58] reported similar results for honey from Kayseri province, detecting Zn levels between 2.2 and 11.0 mg kg^−1^, depending on the distance of the beehives from residential areas. The Ni level in Kayseri honey (0.2 ± 10% mg kg^−1^) measured in present study was consistent with the previously reported results by the same authors (0.2–0.8 mg kg^−1^) [58]. This allows us to agree with the conclusion of Tuzen et al. [54] that trace element concentrations in honey are generally correlated with the degree of trace element contamination of the environment.

All other elements made up below 0.1% of the elemental profile of honey. The toxic elements arsenic, cadmium, nickel and lead were detected in some honeys in quantities below the regulated limits for these elements. The European Commission suggests an acceptable maximum level of 1 mg kg^−1^ for Pb and 0.1 mg kg^−1^ for Cd [59].

We found that some of the honey samples contained palladium, an element which has an exclusively anthropogenic origin. Palladium, together with platinum and rhodium, is used in the production of catalytic converters in cars to reduce the harmful exhaust gases. Due to the abrasion of the catalysts, these elements are emitted into the environment as nanoparticles and can be partly solubilized and mobilized under the action of mobilizing agents present in soil and water. In this way, they are spread in all environmental compartments. Increasingly high concentrations of Pt and Pd have been reported in environmental and biological samples all over the world. To date, only a few studies have evaluated the noble metal content in honey samples. Although platinum is emitted into the environment in higher quantities due to the Pt/Pd > 1 ratio, we registered only palladium in our study [60]. This is not surprising considering that palladium forms more labile complexes than platinum and rhodium and is, therefore, mobilized to a greater extent [61]. After the introduction of catalytic converters in cars, a decrease in Pb levels and an increase in precious metal concentrations in environmental samples were observed [19,62]. Our study showed that these changes in the environment are reflected in the honey composition. In our work, we registered palladium only in individual samples, which suggests the presence of motorways close to some of the beehives. However, the Pd concentration measured in this study (0.09–0.18 mg kg^−1^) was lower than that obtained by Imtara et al. [40] in Palestinian honeys in which the recorded Pd levels were in the range of 0.5–0.9 mg kg^−1^ in all studied samples belonging to different floral and geographical origins. 

Honey displays a wide range of colors, spanning from light white to dark amber. As supported by various studies, the color is closely associated with its mineral composition [4,63]. Dark and amber honeys, exhibit higher levels of major, minor, and trace metals when compared with lighter-colored honeys. Furthermore, researches have indicated that in addition to the mineral composition, the color of honey is also influenced by its floral source. This is attributed to the fact that the morphology and color of pollen grains present in honey can have a significant impact on its overall color [4].

Considering the honey color, our study confirmed its correlation with mineral composition, with the observation that the darker honeys had higher metal contents compared with the paler ones. However, the color is related not only to the mineral content but also to the availability of active pigments, such as carotenoids and flavonoids, and to the presence of products from the decomposition of reducing sugars during storage of the honey [64,65].

### 3.2. Tracing Minerals in Honey to the Floral Source

**Figure 3:**
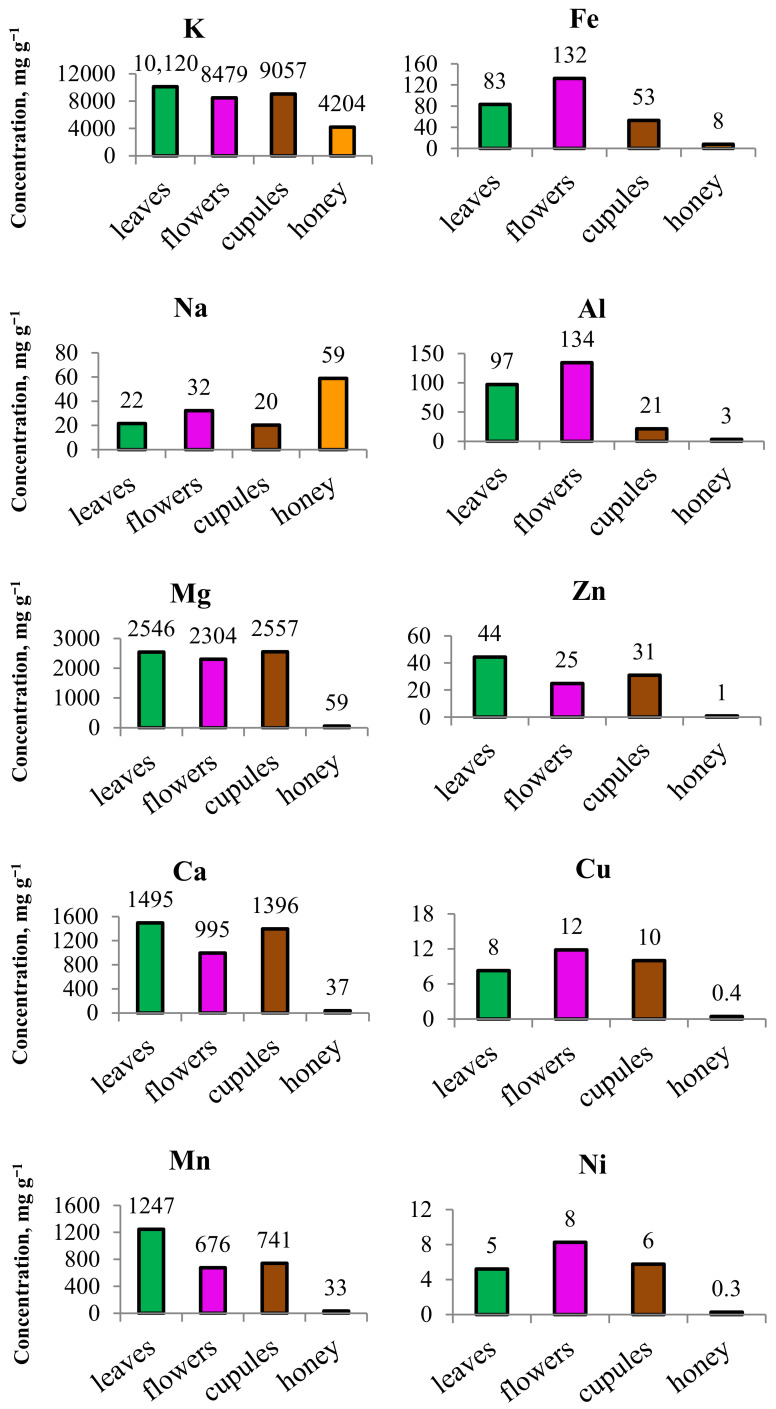
Assessment of mineral concentrations (mg kg^−1^) in chestnut tree (leaves, flowers, cupules) and in chestnut honey for comparative analysis.

As pointed out by many authors, the chemical composition of honey is influenced by the nectar type, climate, soil, and honey-handling practices. Honeys also contain elements specific to the plant that bees use as a food source. The relationship between honey compounds and their origin in botanical species is scarcely studied in the scientific literature. Turski et al. [66], for example, found a high content of kynurenic acid in chestnut honey and correlated it with the high concentration of this compound in the flowers of the chestnut tree. Evidently, different parts of the plant can be considered as precursors of the specific elements in honey, which is why we included an additional step in our study aimed at relating the honey chemical content with the presence of certain elements in selected parts of the plant species. Leaves, flowers and fruit husks (cupules) of a chestnut tree were collected and an analysis of the major and minor element concentrations was performed in order to identify the botanical source of specific elements (Figure 3). The results obtained indicate that, along with the major elements such as potassium, calcium, and sodium, chestnut tree contains significant quantities of manganese, concentrated predominantly in the leaves. This may explain the typically high Mn concentration in chestnut honey. Manganese was the most characteristic element among microelements, followed by iron, zinc, and copper. 

The same observations were reported by Pereira-Lorenzo et al. [67] when analyzing the mineral content in the fruits of Spanish chestnut trees. Furthermore, comparing the minerals present in chestnut tree parts and in honey, we found a positive relationship, namely, the concentration of the elements in honey samples decreased in the same order as in the tree samples, suggesting possible pathway from the tree to bee product composition. An exception to this trend was observed for sodium, which may be due to a different pattern of assimilation. Moreover, we note the similarity in calcium and manganese behavior. Based on the results presented in Figure 3, we can conclude that the investigation on the tree composition is a promising tool that may reveal important knowledge about the model of minerals assimilation and their pathway from the soil to bee products.

### 3.3. Classification of Honeys by Multivariate Techniques

**Figure 4:**
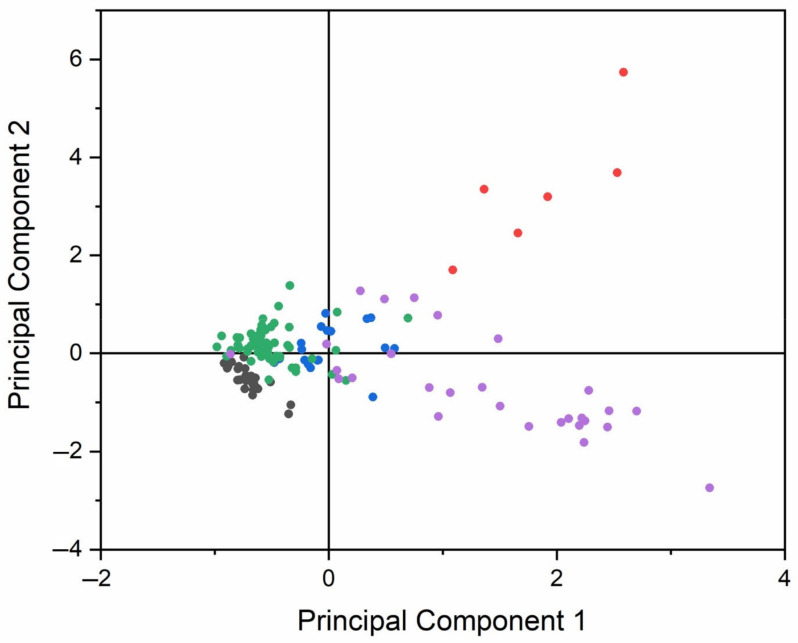
PCA analysis of Slovenian honeys. ChestnutSi (red), ForestSi (violet), LindenSi (blue), FloralSi (green), and AcaciaSi (black).

The PCA analysis of Slovenian honeys is represented in Figure 4. The first and second principal components cover 42.6% and 18.3% of the variance, respectively, giving a total percentage of 60.9%. From Figure 4 we can observe that successful clustering of honey types is achieved. FloralSi, acaciaSi, and lindenSi are centred around the origin and their groups are distinguishable from each other. The acacia and floral honeys are tightly packed into clusters, while the linden honeys are more spread out. The ForestSi cluster is wider and reaches farther from the centre. It partially overlaps with lindenSi close to the origin point.

**Figure 5:**
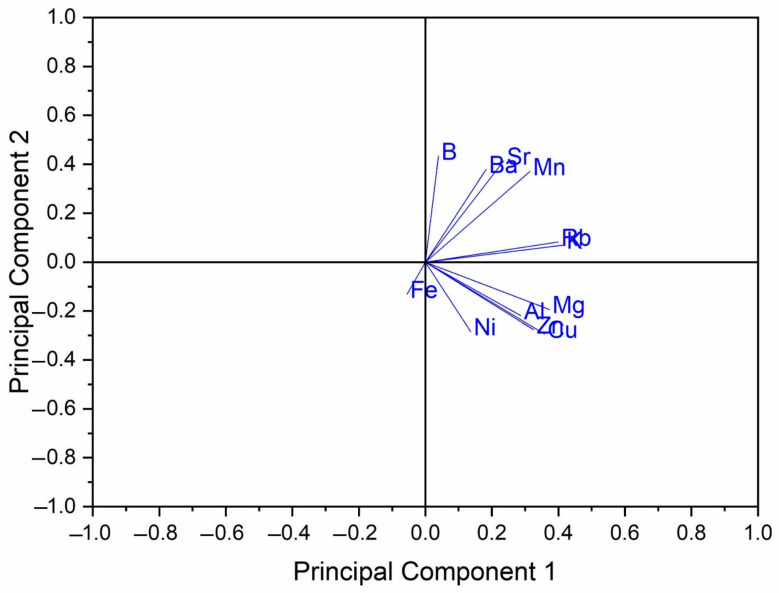
Loading plot of the PCA analysis of Slovenian honeys.

Observation of the loading plot for the elements used in the PCA analysis (Figure 5) shows which elements cause the separation of the honey types. We can observe that the Mn content points towards the direction of chestnut honey samples, which is in accordance with the elemental analysis, in which the Mn content is the highest in chestnut honey.

It is important to highlight that several studies have indicated that machine learning-based analysis and supervised methods exhibit high discrimination performance, particularly in the context of food authentication. These methods include Random Forest (RF), Support Vector Machines (SVM), Feedforward Neural Networks (FNN), Orthogonal Partial Least Squares Discriminant Analysis (OPLS-DA), Linear Discriminant Analysis (LDA) [68,69]. These techniques have been shown to enhance the effectiveness of PCA in various applications [68,70].

Based on the evidence, it becomes apparent that chemometric techniques are a powerful tool for accurately characterizing and classifying honeys based on their sources, encompassing both geographical and botanical aspects [71,72,73,74,75].

## 4. Conclusions

In this study, an assessment of honey composition in view of the different factors influencing the metal content was conducted. One hundred and seventy-three samples from different geographical regions and plant sources were analyzed, giving the possibility to compare the influence of both geographical and botanical origin on honey elemental profiles. We discovered that the mineral content of honey is variable, and factors such as pollen type, environmental conditions and human activity exert different effects. We found that botanical and geographical factors have an impact with specific predominance on particular elements. It was observed that the concentrations of some elements, such as Mn, K, and Ca, depend mostly on the botanical origin, whereas elements such as Na, Mg, and Fe are influenced to a greater extent by environmental factors and could be considered as markers of the geographical origin of honey. An analysis of chestnut tree samples showed a positive relation with honey mineral composition that explained the specific high Mn concentration in chestnut honey.

The results obtained demonstrated that the concentrations of heavy and toxic metals such as Cd, Pb, As, and Ni were below the regulated norms, indicating a good level of quality of all the studied honeys regarding toxicological risk. The presence of Pd in some samples indicates the rise in noble metal levels entering the food chain.

With sufficiently high numbers of honey samples, as in the case of Slovenian honey samples, it is possible to utilize multivariate statistical analysis. Using PCA analysis we showed that five groups of honey (floral, linden, forest, acacia, and chestnut) can be identified by their elemental profile. This makes available another way to classify honey types based on inorganic components, which are present in only micro- or trace quantities.

Besides the specific elemental composition, the total mineralization of a honey, as well as its color, provides preliminary information and could be useful for initial assessment of honey type.

## Figures and Tables

**Table 1:** Geographical and botanical characteristics of studied honey samples.

Honey Variety	Geographical Region	Prevailing Botanical Source	Number of Samples	Year of Collection
AcaciaSi	Slovenia	Acacia (*Robinia pseudoacacia* L.)	26	2016–2018
FlowerSi	Slovenia	Mixed Flower	60	2016–2018
ForestSi	Slovenia	Forest	28	2016–2018
FirSi	Slovenia	Silver Fir *(Abies alba Mill)*	2	2016–2018
ChestnutSi	Slovenia	Chestnut (*Castanea sativa Mill*.)	6	2016–2018
SpruceSi	Slovenia	Spruce (*Picea mariana*)	2	2016–2018
LindenSi	Slovenia	Linden (*Tilia* spp.)	18	2016–2018
LavenderBg	Bulgaria	Lavender (*Lavandula* spp. L.)	1	Spring 2022
AcaciaBg	Bulgaria	Acacia (*Robinia pseudoacacia* L.)	1	Spring 2022
LindenBg	Bulgaria	Linden (*Tilia* spp.)	1	Spring 2022
ForestBg	Bulgaria	Forest	1	Spring 2022
CorianderBg	Bulgaria	Coriander (*Coriandrum sativum* L.)	1	Spring 2022
HoneydewBg	Bulgaria	Honeydew	1	Spring 2022
HerbsBg	Bulgaria	Herbs	1	Spring 2022
ThistleBg	Bulgaria	Thistle (*Cardus nutans*)	2	Spring 2022
FlowerYuksekova	Turkey, Yüksekova	Mixed Flower	1	Spring 2022
FlowerKayseri	Turkey, Kaiseri	Mixed Flower	1	Spring 2022
FlowerMus	Turkey, Muş	Mixed Flower	1	Spring 2022
EuphorbiaMa	Morocco	*Euphorbia* sp.	1	Spring 2022
ZiziphusMa	Morocco	*Ziziphus lotus* L.	1	Spring 2022
FlowerHvar	Hvar, Croatia	Mixed Flower	12	Summer 2022
SageHvar	Hvar, Croatia	Sage (*Salvia officinalis* L.)	2	Summer 2022
RosemaryHvar	Hvar, Croatia	Rosemary (*Rosmarinus officinalis* L.)	2	Summer 2022
FlowerVis	Vis, Croatia	Mixed Flower	1	Summer 2022
RosemaryŠčedro	Ščedro, Croatia	Rosemary (*Salvia rosmarinus* L.)	1	Summer 2022

**Table 2:** Operating conditions for ICP-MS spectrometer.

ICP-MS Spectrometer: Agilent 7900 ICP-MS Spectrometer	
Operating Conditions	
RF power	1.5 kW
Plasma argon flow rate	1.0 L min^−1^
Carrier argon flow rate	0.85 L min^−1^
Make up argon flow rate	0.28 L min^−1^
Sample flow rate	0.2 mL min^−1^
Dwell time per element	0.2 s
Total acquisition time per sample	53 s

## Data Availability

Data is contained within the article or supplementary material.

## Supplementary Materials

The following supporting information can be downloaded at: https://www.mdpi.com/article/10.3390/foods12152826/s1, Figure S1: Concentration of K (mg kg^−1^) in honeys with various plant origin; Figure S2: Concentration of Ca (mg kg^−1^) in honeys with various plant origin; Figure S3: Concentration of Mg (mg kg^−1^) in honeys with various plant origin; Figure S4: Concentration of Na (mg kg^−1^) in honeys with various plant origin; Table S1: Descriptive statistics for major and minor elemental concentration (mg kg^−1^) in honeys, collected in Slovenia, presented by number of samples (n) of each botanical species, mean value (mg kg^−1^), minimum and maximum concentrations (mg kg^−1^), standard deviation (SD), RSD (%), total mineral content (Total) (mg kg^−1^) and percentage (%) of each element related to the total mineral content; Table S2: Descriptive statistics for trace element concentration (mg kg^−1^) in honeys, collected in Slovenia, presented by number of samples (n) of each botanical species, mean value (mg kg^−1^), minimum and maximum concentrations values (mg kg^−1^), standard deviation (SD), RSD (%), and percentage (%) of each element related to the total mineral content; Table S3: Elemental concentration in studied honey samples (mg kg^−1^), total mineral content (mg kg^−1^) and percentage (%) of each element relative to the total mineral content, measured with ICP-MS; Table S4: Elemental concentration (mg kg^−1^), percentage (%) and total mineral content (mg kg^−1^) reported in the literature for honey with various botanical and geographical origin.
