# Supplementary material for: Honey Origin Authentication via Mineral Profiling Combined with Chemometric Approaches
Source: Foods. 2023 Jul 25;12(15):2826. doi: 10.3390/foods12152826 (PMC10417852; doi:10.3390/foods12152826)
Supplement: Supplementary file 1 [file foods-12-02826-s001.zip › Supplement_Figures.pdf]

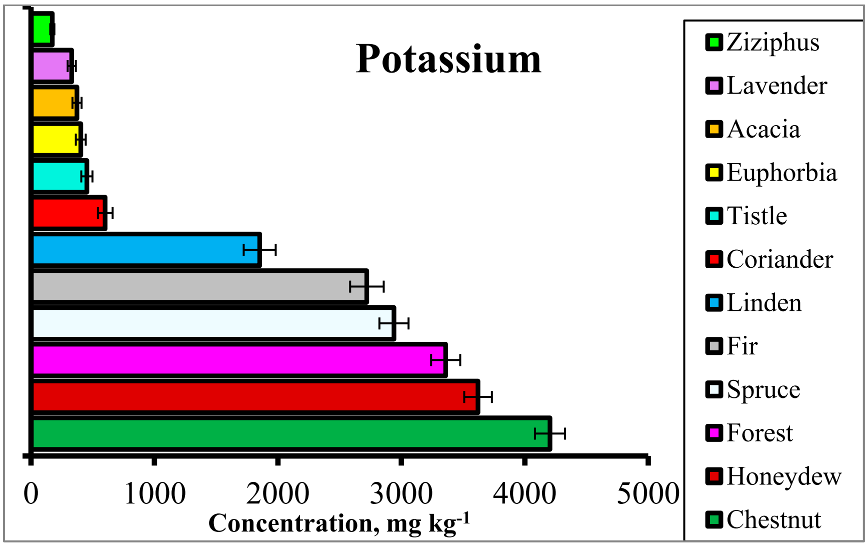

Figure S1. Concentration of K (mg kg^{-1}) in honeys with various plant origin.

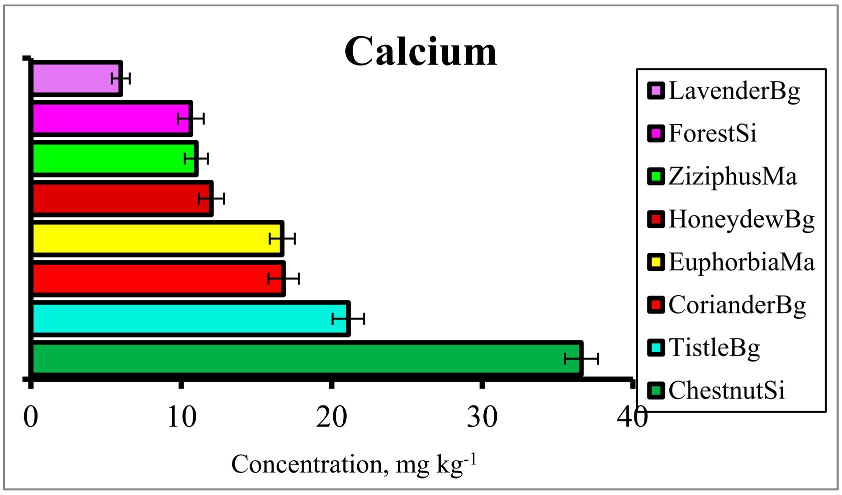

Figure S2. Concentration of Ca (mg kg^{-1}) in honeys with various plant origin.

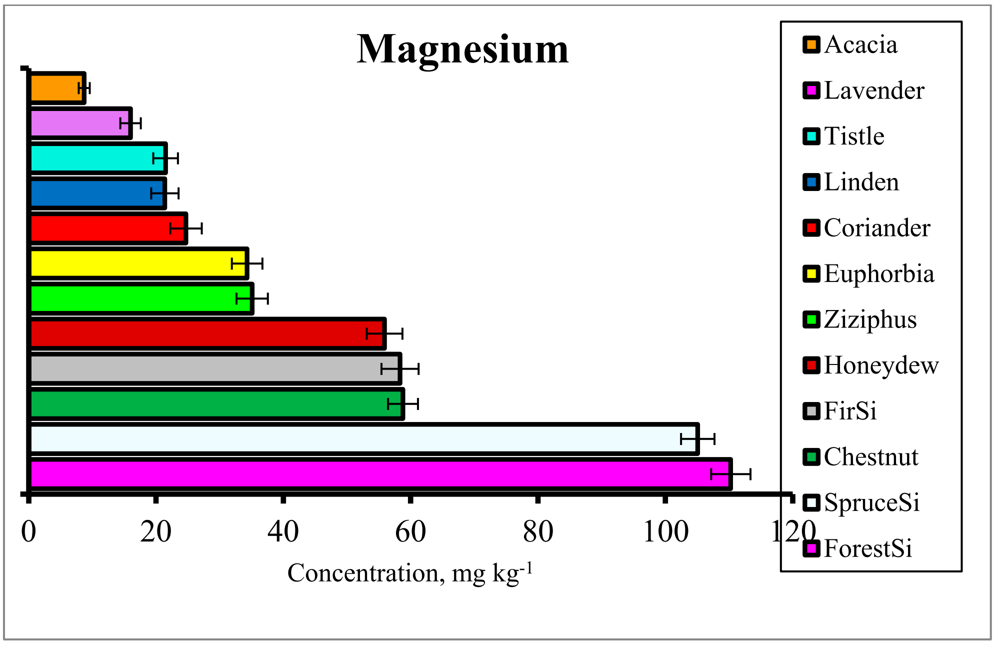

Figure S3. Concentration of Mg (mg kg^{-1}) in honeys with various plant origin**.**

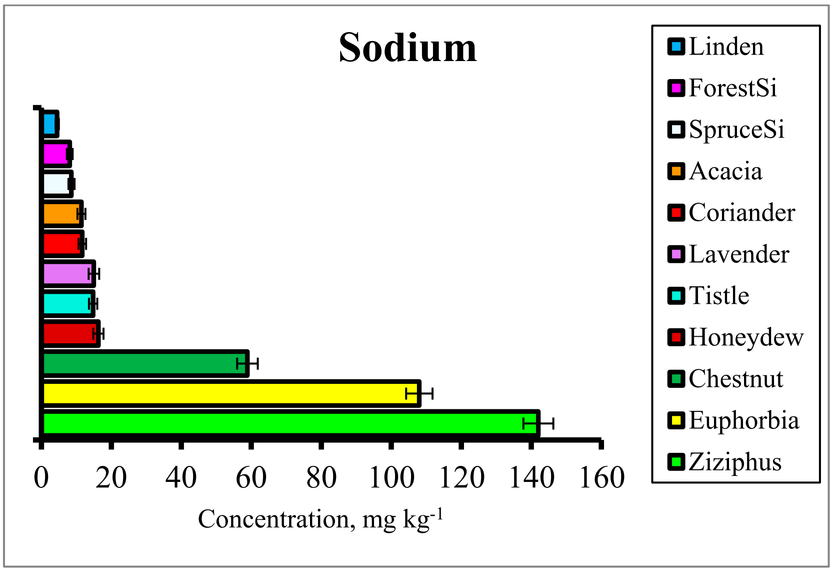

Figure S4. Concentration of Na (mg kg^{-1}) in honeys with various plant origin.
