# Supplementary material for: Honey Origin Authentication via Mineral Profiling Combined with Chemometric Approaches
Source: Foods. 2023 Jul 25;12(15):2826. doi: 10.3390/foods12152826 (PMC10417852; doi:10.3390/foods12152826)
Supplement: Supplementary file 1 [file foods-12-02826-s001.zip › Supplement_Tables.pdf]

Table S1. Descriptive statistics for major and minor elemental concentration (mg kg^{-1}) in honeys, collected in Slovenia, presented by number of samples (n) of each botanical species, mean value (mg kg^{-1}), minimum and maximum concentrations (mg kg^{-1}), standard deviation (SD), RSD (%), total mineral content (Total) (mg kg^{-1}) and percentage (%) of each element related to the total mineral content.

Honey type		K	Ca	Na	Mg	Fe	Zn	Mn	Al	Rb	Total
Spruce	Mean	2939.59	10.49	8.60	105.07	44.63	2.61	5.87	8.97	6.71	3134.02
n=2	SD	29.61	0.34	0.72	1.33	5.59	0.38	0.08	0.08	0.16	
	RSD	1.01	3.24	8.40	1.27	12.53	14.72	1.31	0.92	2.33	
	Min	2918.66	10.25	8.08	104.13	40.68	2.34	5.81	8.92	6.60	
	Max	2960.53	10.73	9.11	106.02	48.59	2.88	5.92	9.03	6.82	
	%	93.80	0.33	0.27	3.35	1.42	0.08	0.19	0.29	0.21	
Silver Fir	Mean	2720.26	6.16	2.36[a]	58.33	53.89	1.39	2.38	13.66	16.88	2878.26
n=2	SD	7.92	1.66		1.55	21.43	0.21	0.00	0.86	0.09	
	RSD	0.29	26.93		2.66	39.76	14.84	0.20	6.30	0.56	
	Min	2714.66	4.99		57.23	38.73	1.24	2.38	13.05	16.82	
	Max	2725.87	7.33		59.43	69.04	1.54	2.39	14.27	16.95	
	%	94.51	0.21	0.08	2.03	1.87	0.05	0.08	0.47	0.59	
Chestnut	Mean	4203.95	36.59	58.90	58.78	7.96	0.89	33.10	3.47	18.70	4423.37
n=6	SD	786.29	7.98	44.19	10.85	11.68	0.26	12.35	4.22	4.92	
	RSD	18.70	21.82	75.02	18.46	146.81	29.31	37.31	121.49	26.33	
	Min	3122.82	27.91	21.17	46.40	0.00	0.59	19.17	1.24	12.62	
	Max	5488.34	48.89	137.47	72.94	0.00	1.30	0.87	12.05	25.65	
	%	95.04	0.83	1.33	1.33	0.18	0.02	0.75	0.08	0.42	
Acacia	Mean	372.43	5.38	11.45	8.72	24.72	0.76	0.36	2.01	0.62	427.68
n=26	SD	110.16	1.03	7.63	1.97	27.12	0.50	0.18	2.92	0.23	
	RSD	29.58	19.22	66.62	22.55	109.72	65.37	49.49	144.70	36.90	
	Min	247.79	4.31	2.06	4.49	0.21	0.22	0.17	0.11	0.37	
	Max	571.90	6.61	19.95	12.39	134.59	2.11	0.87	13.52	1.14	
	%	87.08	1.26	2.68	2.04	5.78	0.18	0.08	0.47	0.15	

Table S1 (continued)

Honey type		K	Ca	Na	Mg	Fe	Zn	Mn	Al	Rb	Total
Linden n=18	Mean	1852.36	15.92	4.53	21.39	19.19	0.88	2.42	2.50	6.80	1927.33
	SD	268.00	1.74	2.09	6.38	19.70	0.53	1.66	2.16	3.26	
	RSD	14.47	10.90	46.03	29.84	102.65	60.40	68.90	2.16	48.02	
	Min	1413.47	13.74	2.18	12.32	1.38	0.05	0.37	0.32	2.27	
	Max	2284.44	17.49	6.14	36.79	70.20	2.08	4.72	7.06	11.65	
	%	96.11	0.83	0.24	1.11	1.00	0.05	0.13	0.13	0.35	
Flower n=60	Mean	1244.67	14.73	17.14	22.65	1.57	0.56	1.21	2.36	2.29	1308.64
	SD	406.58	6.16	7.62	10.09	2.92	0.55	0.75	2.52	1.20	
	RSD	32.67	41.84	44.45	44.56	186.17	98.30	61.45	106.76	52.23	
	Min	347.47	8.48	5.18	10.32	0.01	0.01	0.29	0.64	0.45	
	Max	2224.37	24.63	37.84	62.34	20.63	2.10	3.24	16.29	6.11	
	%	95.11	1.13	1.31	1.73	0.12	0.04	0.09	0.18	0.18	
Forest n=28	Mean	3358.36	10.63	8.12	110.27	3.81	2.46	7.57	9.52	13.29	3525.84
	SD	1054.76	3.18	8.10	72.60	3.60	1.17	4.26	10.42	6.91	
	RSD	31.41	29.87	99.78	65.84	94.42	47.82	56.23	109.45	52.04	
	Min	719.24	7.40	0.06	14.91	0.15	0.38	0.42	0.72	0.92	
	Max	5531.52	15.10	30.69	235.33	16.23	4.53	15.77	52.34	32.97	
	%	95.25	0.30	0.23	3.13	0.11	0.07	0.21	0.27	0.38	

[a] one honey sample measured

Table S2. Descriptive statistics for trace element concentration (mg kg^{-1}) in honeys, collected in Slovenia, presented by number of samples (n) of each botanical species, mean value (mg kg^{-1}), minimum and maximum concentrations values (mg kg^{-1}), standard deviation (SD), RSD (%), and percentage (%) of each element related to the total mineral content.

Honey type		As	Pb	Cr	Co	Ni	Cu	Ag	Cd	Ti	Se	Sr	Pt
Spruce n=2	Mean	-[b]	-	-	0.036	0.094	1.185	-	0.017	0.024	-	0.127	-
	SD	-	-	-	0.011	0.030	0.130	-	0.004	0.018	-	0.028	-
	RSD	-	-	-	29.650	31.720	10.988	-	22.707	73.604	-	22.417	-
	Min	-	-	-	0.029	0.073	1.093	-	0.014	0.012	-	0.107	-
	Max	-	-	-	0.044	0.115	1.277	-	0.020	0.037	-	0.147	-
	%	-	-	-	0.00	0.000	0,04	-	0.000	0.000	-	0.004	-
Silver Fir n=2	Mean	-	-	-	0.019	0.564	2.102	-	0.016	0.098	0.093[a]	0.047	-
	SD	-	-	-	0.003	0.044	0.087	-	0.002	0.039		0.017	-
	RSD	-	-	-	15.038	7.785	4.121	-	10.741	40.076		35.324	-
	Min	-	-	-	0.017	0.533	2.041	-	0.014	0.070		0.036	-
	Max	-	-	-	0.021	0.595	2.163	-	0.017	0.125		0.059	-
	%	-	-	-	0.000	0.020	0.070	-	0.001	0.003	0.00	0.002	-
Chestnut n=6	Mean	0.002[a]	0.014[a]	-	0.025	0.283	0.441	-	0.002	-	-	0.279	-
	SD			-	0.034	0.197	0.150	-	0.000	-	-	0.089	-
	RSD			-	135.343	69.652	34.045	-	13.617	-	-	31.788	-
	Min			-	0.005	0.076	0.285	-	0.002	-	-	0.158	-
	Max			-	0.076	0.468	0.659	-	0.002	-	-	0.388	-
	%	0.00	0.00	-	0.001	0.006	0.010	-	0.000	-	-	0.006	-
Acacia n=26	Mean	-	-	-	0.003	0.639	0.269	-	0.002	0.039	0.236	0.041	-
	SD	-	-	-	0.003	0.450	0.129	-	0.002	0.046	0.414	0.016	-
	RSD	-	-	-	100.774	70.359	47.760	-	101.418	117.557	175.242	38.678	-
	Min	-	-	-	0.000	0.015	0.109	-	0.001	0.000	0.034	0.022	-
	Max	-	-	-	0.008	1.857	0.626	-	0.006	0.163	0.975	0.078	-
	%				0.000	0.151	0.064		0.000	0.009	0.056	0.010	

Table S2 (continued)

Honey type		As	Pb	Cr	Co	Ni	Cu	Ag	Cd	Ti	Se	Sr	Pt
Linden n=18	Mean	-	-	-	0.005	0.722	0.330	-	0.002	0.026	0.144	0.105	-
	SD	-	-	-	0.005	0.534	0.246	-	0.002	0.022	0.274	0.053	-
	RSD	-	-	-	107.144	73.925	74.498	-	96.494	0.022	190.315	50.589	-
	Min	-	-	-	0.000	0.047	0.078	-	0.001	0.006	0.034	0.033	-
	Max	-	-	-	0.014	2.079	1.043	-	0.005	0.076	0.764	0.253	-
	%	-	-	-	0.000	0.038	0.017	-	0.000	0.000	0.008	0.006	-
Flower n=60	Mean	0.002	0.113	0.260	0.103	0.529	0.261	0.083	0.006	0.027	-	0.066	-
	SD	0.001	0.181	0.086	0.645	0.335	0.175	0.080	0.008	0.012	-	0.047	-
	RSD	50.050	159.934	33.234	625.750	63.384	67.122	95.983	126.048	43.780	-	70.822	-
	Min	0.000	0.001	0.010	0.000	0.124	0.025	0.029	0.001	0.002	-	0.016	-
	Max	0.006	0.433	0.334	4.234	0.938	0.941	0.224	0.012	0.050	-	0.268	-
	%	0.000	0.008	0.019	0.008	0.039	0.039	0.006	0.000	0.002	-	0.005	-
Forest n=28	Mean	0.001	0.040	-	0.041	0.584	0.948	0.006	0.017	-	0.072	0.107	-
	SD	0.001	0.048	-	0.035	0.452	0.592	0.008	0.015	-	0.000	0.063	-
	RSD	62.041	119.619	-	84.858	77.453	62.478	136.653	91.166	-	0.000	58.762	-
	Min	0.000	0.000	-	0.004	0.021	0.015	0.001	0.001	-	0.000	0.003	-
	Max	0.003	0.201	-	0.131	1.514	2.557	0.016	0.048	-	0.000	0.253	-
	%	0.000	0.001	-	0.001	0.016	0.027	0.000	0.000	-	0.002	0.003	-

[a] one honey sample measured

[b] below DLs

Table S3. Elemental concentration in studied honey samples (mg kg^{-1}), total mineral content (mg kg^{-1}) and percentage (%) of each element relative to the total mineral content, measured with ICP-MS

Honey type	Elements																		Total
	K	Ca	Na	Mg	Fe	Zn	Mn	As	Pb	Al	Cr	Co	Ni	Cu	Ag	Cd	Pd	Pt	
ChestnutSi	4203.95^{a*}	36.58^{a}	58.90^{c}	$58.78^{c,d}$	7.96	0.89	33.10	0.002	0.01	3.47	$-^{**}$	0.03	0.28	0.44	-	0.002	-	-	4423.37^{a}
%	95.04	0.83	1.33	1.33	0.18	0.02	0.75	-	-	0.08	-	0.00	0.01	0.01	-		-	-	
LindenSi	1852.36^{g}	15.92^{k}	$4.53^{k,l}$	$21.39^{j,k}$	19.19	0.88	2.42	-	-	2.50	-	0.005	0.72	0.33	-	0.002	-	-	1927.33^{f}
%	96.11	0.83	0.24	1.11	1.00	0.05	0.13	-	-	0.13	-	0.00	0.04	0.02	-	0.00	-	-	
SpruceSi	2939.59^{d}	10.49^{k}	$8.60^{j,k}$	105.07^{a}	44.63	2.61	5.87	-	-	8.97	-	0.04	0.09	1.19	-	0.02	-	-	3134.02^{c}
%	93.80	0.33	0.27	3.35	1.42	0.08	0.19	-	-	0.29	-	0.00	0.00	0.04	-	0.00	-	-	
AcaciaSi	372.43^{m}	5.38^{k}	$11.45^{i,j}$	8.72^{n}	24.72	0.76	0.36	-	-	2.02	-	0.003	0.64	0.27	-	0.002	-	-	$427.68^{k,l,m}$
%	87.08	1.26	2.68	2.04	5.78	0.18	0.08	-	-	0.47	-		0.15	0.06	-	0.00	-	-	
ForestSi	3358.36^{c}	10.63^{c}	$8.12^{j,k}$	110.27^{a}	3.81	2.46	7.57	0.001	0.04	9.52	-	0.04	0.58	0.95	0.006	-	-	-	3525.84^{b}
%	95.25	0.30	0.23	3.13	0.11	0.07	0.21	0.00	0.00	0.27	-	0.00	0.02	0.03	0.00	0.00	-	-	
FirSi	2720.26^{e}	6.16^{k}	2.36^{l}	$58.33^{c,d}$	53.89	1.39	2.38	-	-	13.66	-	0.02	0.56	2.10	-	0.02	-	-	2878.26^{d}
%	94.51	0.21	0.08	2.03	1.87	0.05	0.08	-	-	0.47	-	0.001	0.02	0.07	-	0.001	-	-	
FlowerSi	1244.67^{i}	14.73^{b}	17.14^{g}	$22.65^{j,k}$	1.57	0.56	1.21	0.002	0.11	2.36	0.26	0.10	0.53	0.26	0.08	0.006	-	-	1308.64^{h}
%	95.11	1.13	1.31	1.73	0.12	0.04	0.09	0.00	0.01	0.18	0.02	0.01	0.04	0.04	0.01	0.00	-	-	

Table S3 (continued)

Honey type	Elements																		Total
	**K**	**Ca**	**Na**	**Mg**	**Fe**	**Zn**	**Mn**	**As**	**Pb**	**Al**	**Cr**	**Co**	**Ni**	**Cu**	**Ag**	**Cd**	**Pd**	**Pt**	
LavenderBg	329^{m,n}	5.98^{j}	15.0^{g,h,i}	16.0^{l,m}	0.67	-	0.63	-	-	-	0.13	-	-	0.24	-	-	-	-	367.80^{m}
%	89	1.63	4.10	4.40	0.18	-	0.17	-	-	-	0.04	-	-	0.06	-	-	-	-	
AcaciaBg	246^{m,n}	2.68^{k}	11.3^{i,j}	6.81^{n}	0.48	-	0.17	-	0.15	-	0.12	-	-	0.16	-	-	0.15	-	267.80^{m}
%	92	1.0	4.20	2.54	0.18	-	0.06	-	0.05	-	0.05	-	-	0.06	-	-	0.05	-	
LindenBg	1510^{h}	9.69^{h,i}	15.9^{g,h,i}	37.9^{f,g}	1.74	-	3.88	-	0.09	-	0.11	-	-	0.38	-	-	-	-	1579.40^{g}
%	96	0.61	1.00	2.40	0.11	-	0.25	-	0.01	-	0.01	-	-	0.02	-	-	-	-	
ForestBg	915^{j}	26.50^{d}	12.50^{h,i,j}	43.20	0.82	0.17	0.69	-	-	-	0.10	-	-	0.23	-	-	0.18	-	999.50^{i}
%	92	2.60	1.3	4.3	0.08	0.02	0.07	-	-	-	0.01	-	-	0.02	-	-	0.02	-	
CorianderBg	601^{k}	16.8^{f}	11.7^{i,j}	24.7^{j}	1.02	0.04	0.23	-	-	-	-	-	-	0.15	-	-	0.13	-	656.30^{j,k}
%	92	2.6	1.8	3.8	0.16	0.01	0.04	-	-	-	-	-	-	0.02	-	-	0.02	-	
HoneydewBg	3621^{b}	12.0^{g,h}	16.30^{g,h}	55.9^{d}	3.62	-	4.91	-	-	-	-	-	-	1.08	5.94	-	-	-	3720.90^{b}
%	97	0.30	0.40	1.50	0.10	-	0.13	-	-	-	-	-	-	0.03	0.16	-	-	-	
HerbsBg	685^{k}	27.30^{d}	15.10^{g,h,i}	41.70^{e,f}	-	-	0.25	-	0.08	-	-	-	-	0.16	4.63	-	-	-	774.20^{j}
%	88	3.50	2.0	5.40	-	-	0.03	-	0.01	-	-	-	-	0.02	0.60	-	-	-	
TistleBg	621.50^{k}	21.10^{e}	12.0^{h,i,j}	30.34^{h,i}	7.02	1.34	0.35	-	-	-	-	-	-	0.21	-	-	0.09	-	693.90^{j}
%	89.57	3.04	1.7	4.37	1.01	0.19	0.05	-	-	-	-	-	-	0.03	-	-	0.01	-	

Table S3 (continued)

Honey type	Elements																		Total
	K	Ca	Na	Mg	Fe	Zn	Mn	As	Pb	Al	Cr	Co	Ni	Cu	Ag	Cd	Pd	Pt	
FlowerYuksek	377[m]	11.60[g,h]	18.90[g]	13.70[m]	3.01	1.48	0.46	-	-	-	-	-	-	0.18	-	-	-	-	426.70[k,l,m]
%	88	2.70	4.40	3.20	0.70	0.35	0.11	-	-	-	-	-	-	0.04	-	-	-	-	
FlowerKayseri	559[k,l]	10.40[h]	36.30[e]	20.70[j,k,l]	2.33	3.50	0.28	-	-	-	-	-	0.19	0.41	-	-	0.15	-	633.40[j,k]
%	88	1.60	5.70	3.30	0.37	0.55	0.04	-	-	-	-	-	0.03	0.06	-	-	0.02	-	
FlowerMus	275[m,n]	7.46[i,j]	28.0[f]	18.4[k,l,m]	4.06	3.82	0.33	-	-	-	-	-	-	0.16	-	-	0.12	-	336.90[m]
%	82	2.22	8.30	5.40	1.21	1.13	0.10	-	-	-	-	-	-	0.05	-	-	0.03	-	
EuphorbiaMa	403[l,m]	16.70[f]	108[b]	34.3[g,h,i]	4.30	0.65	0.69	-	-	0.41	-	-	-	0.13	20.10	-	-	-	588.10[j,k,l]
%	68	2.80	18	5.80	0.73	0.11	0.12	-	-	0.07	-	-	-	0.02	3.40	-	-	-	
ZiziphusMa	172[n]	11.0[h]	142[a]	35.10[g,h]	1.49	-	0.10	-	-	-	-	-	-	-	-	-	0.16	-	361.50[l,m]
%	47.6	3.05	39.16	9.70	0.41	-	0.03	-	-	-	-	-	-	-	-	-	0.05	-	
FlowerHvar	2111.4[f]	12.22[g,h]	33.44[e]	61.87[c]	3.94	2.45	1.35	-	-	2.14	-	0.02	0.20	0.96	-	-	-	-	2230.0[g]
%	95	0.5	1.5	2.8	0.18	0.11	0.06	-	-	0.1	-	0.00	0.01	0.04	-	-	-	-	
SageHvar	2044.8[f]	14.1[f,g]	41.3[d]	69.3[b]	2.8	0.9	1.0	-	-	-	-	-	0.8	1.0	-	-	-	-	2176.2[e]
%	94	0.6	1.9	3.2	0.13	0.04	0.05	-	-	-	-	-	0.04	0.05	-	-	-	-	
FlowerVic	1334[i]	7.79[i,j]	41.2[d]	27.7[i]	0.97	15.6	1.2	-	-	1.58	-	-	0.16	0.75	-	-	-	-	1430.87[g,h]
%	93	0.54	2.9	1.9	0.07	1.1	0.09	-	-	0.11	-	-	0.01	0.05	-	-	-	-	

Table S3 (continued)

Honey type	Elements																		Total
	**K**	**Ca**	**Na**	**Mg**	**Fe**	**Zn**	**Mn**	**As**	**Pb**	**Al**	**Cr**	**Co**	**Ni**	**Cu**	**Ag**	**Cd**	**Pd**	**Pt**	
RosemaryŠčed	354^{m}	2.02^{k}	25.3^{f}	5.20^{n}	0.37	0.38	0.12	-	-	-	-	-	0.32	-	-	-	-	-	$387.33^{l,m}$
%	91.29	0.52	6.54	1.34	0.10	0.10	0.03	-	-	-	-	-	0.08	-	-	-	-	-	

* Values within one column labeled with different letters are significantly different based on the results of Duncan's test. (P <0.05)

** Below DLs

Table S4. Elemental **c**oncentration (mg kg^{-1}), percentage (%) and total mineral content (mg kg^{-1}) reported in the literature for honey with various botanical and geographical origin

Honey type	Country	Elements													Total[b]	Reference
		K	Ca	Na	Mg	Fe	Zn	Mn	Al	Cu	S	Cl	Rb	Ni		
Acacia	Slovenia	313	19.10	-[a]	-	-	-	3.09	-	-	48.6	55.4	0.84	-	440.03	[55]
	%	71.13	4.34	-	-	-	-	0.70	-	-	11.04	12.59	0.19			
	Malaysia	1292.28	106.83	173.33	23.27	162.4	12.4	-	-	-	-	-	-	-	1770.54	[71]
	%	72.98	6.03	9.78	1.31	9.17	0.70	-	-	-	-	-	-	-		
	Poland	166	48.8	13	10.1	1.2	4.1	0.5	-	0.1	-	-	-	-	243.8	[41]
	%	68.09	20.01	5.33	4.14	0.49	1.68	0.20	-	0.04	-	-	-	-		
	Bulgaria	126	32	8.11	6	0.83	0.22	0.11	0.8	-	-	-	-	-	174.07	[49]
	%	72.38	18.38	4.65	3.44	0.47	0.12	0.06	0.45	-	-	-	-	-		
	Hungary	327.9	28.1	23.5	10.4	-	2.6	3.3	1.6	-	-	-	-	-	397.4	[3]
	%	82.51	7.07	5.91	2.61	-	0.65	0.83	0.40	-	-	-	-	-		
	China	174.67	18.73	5.3	4.97	0.61	-	0.07	1.7	0.06	-	-	-	-	206.118	[8]
	%	84.74	9.08	2.58	2.40	0.29	-	0.03	0.82	0.03						
	Turkey	267.95	53.5	-	-	1.3	-	0.17	-	0.26	-	-	-	-	323.23	[46]
	%	82.9	16.57	-	-	0.40	-	0.05	-	0.08	-	-	-	-		
	Serbia	239	32.8	-	6.2	1.24	0.78	-	-	-	-	-	-	-	280.02	[72]
	%	85.35	11.71	-	2.21	0.44	0.27	-	-	-	-	-	-	-		
Linden	Slovenia	1670	63.6	-	-	-	-	2.86	-	-	27.1	339	4.04	-	2106.6	[55]
	%	79.27	3.01	-	-	-	-	0.13	-	-	1.28	16.09	0.19			
	Poland	393	39.7	18.9	16	2.5	8.4	1.1	-	0.3	-	-	-	-	479.9	[41]
	%	81.89	8.27	3.93	3.33	0.52	1.75	0.22	-	0.06	-	-	-	-		

Table S4 (continued)

Honey type	Country	Elements													Total	Reference
		**K**	**Ca**	**Na**	**Mg**	**Fe**	**Zn**	**Mn**	**Al**	**Cu**	**S**	**Cl**	**Rb**	**Ni**		
Linden	Bulgaria	792	77	7.5	21	1.62	1.04	2.45	0.39	-	-	-	-	-	903	[49]
	%	87.70	8.52	0.83	2.32	0.17	0.11	0.27	0.04	-	-	-	-	-		
	Hungary	1327.5	111.9	32.6	22.4	-	1.7	1.3	1.3	-	-	-	-	-	1498.7	[3]
	%	88.57	7.46	2.17	1.49	-	0.11	0.08	0.08	-	-	-	-	-		
Chestnut	Slovenia	3290	143	-	-	-	-	20.6	-	-	52.6	226	14.8	-	3747	[55]
	%	87.80	3.81	-	-	-	-	0.54	-	-	1.40	6.03	0.39			
	Poland	709	55	7.8	49.3	1.4	0.7	0.8	-	0.6	-	-	-	-	824.6	[41]
	%	85.98	6.66	0.94	5.97	0.16	0.08	0.09	-	0.07	-	-	-	-		
	Bulgaria	1628	66	9.55	16	0.59	0.2	3.73	0.64	-	-	-	-	-	1724.71	[49]
	%	94.39	3.82	0.55	0.92	0.03	0.01	0.21	0.03	-	-	-	-	-		
	Hungary	2466.3	161.4	43.2	52.3	-	1.2	11.9	1.5	-	-	-	-	-	2737.8	[3]
	%	90.08	5.89	1.57	1.91	-	0.04	0.43	0.05	-	-	-	-	-		
	Turkey	1612.7	104.4	-	-	1.9	-	5.9	-	0.39	-	-	-	-	1725.29	[46]
	%	93.47	6.05	-	-	0.11	-	0.34		0.02	-	-	-	-		
	Spain	2343	159	54	119	7	2.3	-	-	1.9	-	-	-	-	2686.2	[47]
	%	87.22	5.92	2.01	4.43	0.26	0.08	-	-	0.07	-	-	-	-		
Honeydew	Poland	621	53.4	20	45.2	2.7	5.2	3.5	-	0.9	-	-	-	-	751.9	[41]
	%	82.59	7.10	2.65	6.01	0.35	0.69	0.46	-	0.11	-	-	-	-		
	Bulgaria	1121	92	16.3	97	1.73	0.47	12.7	0.47	-	-	-	-	-	1341.67	[49]
	%	83.55	6.86	1.21	7.23	0.13	0.03	0.95	0.03	-	-	-	-	-		
	Hungary	2069.1	134.4	62.6	118.7	-	2.3	5.5	1	-	-	-	-	-	2393.6	[3]
	%	86.44	5.61	2.61	4.96	-	0.09	0.23	0.04	-	-	-	-	-		

Table S4. (continued)

Honey type	Country	Elements													Total	Reference
		K	Ca	Na	Mg	Fe	Zn	Mn	Al	Cu	S	Cl	Sr	Ni		
Honeydew	Morocco	2388.1	-	-	230.05	32.9	1.75	2.65	-	1.7	-	-	-	-	2657.19	[56]
	%	89.87			8.65	1.23	0.06	0.10		0.06						
	New Zeland	3640	7.21	8.51	86.3	3.39	2.46	0.43	5.42	0.18	-	-	-	0.65	3754.55	[15]
	%	96.94	0.19	0.22	2.29	0.09	0.06	0.01	0.14	0.005				0.02		
	Morocco	1298	74.33	122.67	45.17	7.71	2.49	-	22.8	-	-	-	-	-	1573.18	[37]
	%	82.50	4.72	7.79	2.87	0.49	0.15		1.44							
	Spain	2397	147	39	213	5	2.2	-	-	1.6	-	-	-	-	2804.8	[47]
	%	85.46	5.24	1.39	7.59	0.17	0.08			0.06						
Lavender	Morocco	605	65	243.25	32	3.27	3.17	-	13.6	-	-	-	-	-	965.33	[37]
	%	62.67	6.73	25.20	3.31	0.34	0.33		1.41							
	Turkey	120.5	117.8	-	-	1.2	-	0.07	-	0.26	-	-	-	-	239.83	[46]
	%	50.24	49.11			0.50		0.02		0.11						
Coriandrum	Bulgaria	564	44	14.2	7.1	1.33	0.27	0.15	0.49	-	-	-	-	-	631.54	[49]
	%	89.30	6.977	2.25	1.12	0.21	0.04	0.02	0.07							
Forest	Hungary	1892.7	121.5	46.1	71.5	-	4.2	3	1.8	-	-	-	-	-	2140.8	[3]
	%	88.41	5.67	2.15	3.34		0.19	0.14	0.08							
Eucaliptus	Spain	1094	184.4	168.6	36.61	-	5.43	7.427	-	0.578	-	-	0.41	-	1497.46	[26]
	%	73.05	12.31	11.26	2.44		0.36	0.50		0.04			0.03			
	Morocco	336.06	-	-	27.35	12.55	4.36	6.74	-	1.33	-	-	-	-	388.41	[56]
	%	86.52			7.04	3.23	1.12	1.73		0.34						
	Morocco	695.83	103.33	308	29.42	3.82	4.18	-	13.3	-	-	-	-	-	1157.9	[37]
	%	60.09	8.92	26.59	2.54	0.32	0.36		1.15							

Table S4 (continued)

Honey type	Country	Elements													Total	Reference
		K	Ca	Na	Mg	Fe	Zn	Mn	Al	Cu	S	Cl	Sr	Ni		
Eucaliptus	Spain	1010	123	99	20	11	1.6	-	-	2.3	-	-	-	-	1266.9	[47]
	%	79.72	9.71	7.81	1.58	0.87	0.13	-	-	0.18	-	-	-	-		
Tistle	New Zeland	200	20.6	10.4	9.41	1.9	1.05	0.18	-	0.13	-	-	-	0.21	243.88	[15]
	%	82.00	8.44	4.26	3.85	0.78	0.43	0.07		0.05				0.09		
Rape	Poland	190	51.3	11.2	18.9	3	2.1	0.6	-	0.1	-	-	-	-	277.2	[41]
	%	68.54	18.50	4.04	6.81	1.08	0.75	0.21	-	0.03	-	-	-	-		
	Hungary	399.4	68.6	22.8	19.2	-	2.3	0.9	2.2	-	-	-	-	-	515.4	[3]
	%	77.49	13.31	4.42	3.72	-	0.44	0.17	0.42	-	-	-	-	-		
	Poland	356.7	55.3	23.9	10.6	-	1.08	1.08	1.18	0.174	-	-	-	0.07	450.09	[45]
	%	79.25	12.28	5.31	2.35	-	0.24	0.24	0.26	0.04	-	-	-	0.02		
Thyme	New Zeland	335	39	15	9.96	1.33	22.3	0.18	0.9	-	-	-	-	0.02	423.69	[15]
	%	79.06	9.20	3.54	2.35	0.31	5.26	0.04	0.21	-	-	-	-	0.005		
	Morocco	554.17	68.33	153.17	22.5	7.84	3.91	-	17.6	-	-	-	-	-	827.52	[37]
	%	66.96	8.25	18.51	2.72	0.94	0.47		2.12	-	-	-	-	-		
	Spain	1502	98.1	36	40.7	6.05	1.39	2.1	36.7	0.69	-	-	-	0.11	1723.84	[73]
	%	87.13	5.69	2.08	2.36	0.35	0.08	0.12	2.12	0.04	-	-	-	0.006		
	Spain	716.68	185.3	388.7	78.48	6.19	1.78	1.2	17.53	2.44	-	-	0.66	0.13	1399.09	[74]
	%	51.22	13.24	27.78	5.61	0.44	0.13	0.08	1.25	0.17			0.04	0.009		
	Palestina	471.63	96.95	68.47	22.98	4.24	25.2	--	-	0.7	-	-	-	-	690.17	[40]
	%	68.33	14.04	9.92	3.33	0.61	3.65	-	-	0.10	-	-	-	-		

Table S4 (continued)

Honey type	Country	Elements													Total	Reference
		K	Ca	Na	Mg	Fe	Zn	Mn	Al	Cu	S	Cl	Sr	Ni		
Avocado	Spain	1778	113	279	136	9.19	5.65	1.14	9.91	4.18	-	-	0.41	0.33	2336.81	[38]
	%	76.08	4.83	11.94	5.82	0.39	0.24	0.05	0.42	0.18	-	-	0.02	0.01		
	Izrael	1153.7	84.5	61.6	64	2.9	4.1	-	-	0.66	-	-	-	-	1371.46	[75]
	%	84.12	6.16	4.49	4.66	0.21	0.30	-	-	0.05	-	-	-	-		
Rosemary	Spain	754.2	140	36.8	37.82	-	4.49	0.819	-	-	-	-	0.53	-	974.66	[26]
	%	77.38	14.36	3.77	3.88		0.46	0.08	-	-	-	-	0.05	-		
	Morocco	375	24	45	10	1.42	2.46	-	8.66	-	-	-	-	-	466.54	[37]
	%	80.38	5.14	9.64	2.14	0.30	0.53	-	1.86	-	-	-	-	-		
Clover	Egipt	213	-	478	300	58	9.3	0.5	-	1.75	-	-	3.2	4.1	1067.85	[39]
	%	19.94		44.76	28.09	5.43	0.87	0.04	-	0.16	-	-	0.30	0.38		
	New Zeland	226	69.2	17.4	13.3	1.59	1.9	0.46	0.21	0.22				0.43	330.71	[15]
	%	68.33	20.92	5.26	4.02	0.48	0.57	0.14	0.06	0.07				0.13		
Sesame	Egipt	1500	-	378	102	202	7.2	1.7	-	1.7	-	-	0.5	1.25	2194.35	[39]
	%	68.35		17.23	4.64	9.20	0.32	0.07	-	0.08	-	-	0.02	0.06		
Pineapple	Malaysia	473.68	74.6	111.29	36.63	173.6	11.73	-	-	-	-	-	-	-	881.53	[71]
	%	53.73	8.46	12.62	4.15	19.69	1.33	-	-	-	-	-	-	-		
Orange	Spain	455	51.52	11.69	15.35	-	4.05	0.133	-	-	-	-	0.66	-	538.412	[26]
	%	84.51	9.57	2.17	2.85	-	0.75	0.03	-	-	-	-	0.12	-		
	Egipt	528	-	412	225	80	5	0.5	-	1.0	-	-	0.5	1.7	1253.7	[39]
	%	42.11	-	32.86	17.94	6.38	0.40	0.04	-	0.08	-	-	0.04	0.14		

Table S4 (continued)

Honey type	Country	Elements													Total	Reference
		K	Ca	Na	Mg	Fe	Zn	Mn	Al	Cu	S	Cl	Sr	Ni		
Citrus	Morocco	133.174	-	-	26.554	7.97	0.78	0.56	-	0.82	-	-	-	-	169.865	[56]
	%	78.40	-	-	15.63	4.69	0.46	0.33	-	0.48	-	-	-	-		
	Morocco	458.2	38.4	78.59	8.59	2.2	3.63	-	10.2	-	-	-	-	-	599.84	[37]
	%	76.39	6.40	13.10	1.43	0.37	0.60	-	1.70	-	-	-	-	-		
	Palestina	203.92	92.01	35.19	3.8	30.26	1.27	-	-	0.95	-	-	-	-	367.4	[40]
	%	55.50	25.04	9.58	1.03	8.23	0.34	-	-	0.26	-	-	-	-		
Heather	Spain	1771	263	97.66	61.34	-	3.21	4.54	-	0.81	-	-	0.27	-	2201.83	[26]
	%	80.43	11.94	4.43	2.79		0.15	0.21		0.04			0.01			
	Turkey	512.8	63.85	-	-	9.48	-	0.71	-	0.45	-	-	-	-	587.28	[46]
	%	87.31	10.87	-	-	1.61		0.12	-	0.08	-	-	-	-		
	Spain	1135	113	151	25	6	1.6	-	-	1.8	-	-	-	-	1433.4	[47]
	%	79.18	7.88	10.53	1.74	0.42	0.11	-	-	0.13	-	-	-	-		
Carob	Morocco	749.17	135.33	223.67	67.83	4.86	3.05	-	21.45	-	-	-	-	-	1205.36	[37]
	%	62.15	11.23	18.56	5.63	0.40	0.25	-	1.78	-	-	-	-	-		
Multifloral	Poland	344	38.9	26.2	15.5	1.8	3.8	0.9	-	0.2	-	-	-	-	431.3	[41]
	%	79.75	9.01	6.07	3.59	0.41	0.88	0.20		0.04	-	-	-	-		
	Hungary	696.5	110.9	17.4	29.5	-	3.1	2.7	1.4	-	-	-	-	-	861.5	[3]
	%	80.85	12.87	2.02	3.42	-	0.36	0.31	0.16	-	-	-	-	-		
Schisandra	China	219.094	15.659	4.986	5.556	0.534	0	0.072	0.32	0.075	-	-	-	-	246.29	[8]
	%	88.96	6.36	2.02	2.26	0.22	-	0.03	0.13	0.03	-	-	-	-		

Table S4 (continued)

Honey type	Country	Elements													Total	Reference
		K	Ca	Na	Mg	Fe	Zn	Mn	Al	Cu	S	Cl	Sr	Ni		
Vicia dichroantha	China	111.49	9.709	7.097	4.077	0.347	-	0.04	0.46	0.038	-	-	-	-	133.261	[8]
	%	83.66	7.29	5.33	3.06	0.26	-	0.03	0.35	0.03	-	-	-	-		
Astragalus sinicus	China	437.196	36.862	11.589	15.78	0.975	-	0.179	0.75	0.088	-	-	-	-	503.422	[8]
	%	86.84	7.32	2.30	3.13	0.19	-	0.03	0.15	0.017	-	-	-	-		
Euphorbia resinifera	Morocco	641	71.8	127.4	32.6	6.55	2.41	-	17.95	-	-	-	-	-	899.71	[37]
	%	71.24	7.98	14.16	3.62	0.73	0.27	-	1.99	-	-	-	-	-		
Euphorbia echinus	Morocco	575	51	92	24	5.92	4.02	-	15.7	-	-	-	-	-	767.61	[37]
	%	74.91	6.64	11.98	3.12	0.77	0.52	-	2.04	-	-	-	-	-		
Ziziphus L.	Morocco	1268.33	63.33	146.17	30.17	9.34	2.48	-	22.8	-	-	-	-	-	1542.62	[37]
	%	82.22	4.10	9.47	1.95	0.60	0.16	-	1.48	-	-	-	-	-		
Ziziphus jujuba	China	1006.95	89.67	19.28	11.83	1.37	1.03	0.15	1.15	0.237	-	-	-	-	1131.7	[8]
	%	88.98	7.92	1.70	1.04	0.12	0.09	0.01	0.10	0.02	-	-	-	-		
Zisiphus spina christi	Palestina	476.4	94.56	115.04	34.48	3.93	2.48	-	-	1.02	-	-	-	-	727.91	[40]
	%	65.45	13.00	15.80	4.73	0.53	0.34	-	-	0.14	-	-	-	-		
Oak	Turkey	1010.36	111.9	-	-	1.86	-	2.06	-	0.68	-	-	-	-	1136.9	[46]
	%	88.87	9.84	-	-	0.16	-	1.06	-	0.06	-	-	-	-		
Blackberry	Spain	1302	106	30	100	3	1.9	-	-	1.8	-	-	-	-	1544.7	[47]
	%	84.29	6.87	1.949	6.48	0.20	0.13	-	-	0.12	-	-	-	-		
Meadow	Serbia	239	32.8	-	6.2	1.24	0.78	-	-	-	-	-	-	-	627.73	[72]
	%	85.35	11.71	-	2.21	0.44	0.27	-	-	-	-	-	-	-		
Sunflower	Serbia	590	162	-	24.5	0.89	1.73	-	-	-	-	-	-	-	779.12	[72]
	%	75.72	20.79	-	3.14	0.11	0.22	-	-	-	-	-	-	-		

[a] Not analysed

[b] The total mineral content is calculated based on the data presented in the cited article
